# A portable prototype magnetometer to differentiate ischemic and non-ischemic heart disease in patients with chest pain

**DOI:** 10.1371/journal.pone.0191241

**Published:** 2018-01-19

**Authors:** Shima Ghasemi-Roudsari, Abbas Al-Shimary, Benjamin Varcoe, Rowena Byrom, Lorraine Kearney, Mark Kearney

**Affiliations:** 1 Department of Physics and Astronomy, University of Leeds, Leeds, United Kingdom; 2 Division of Cardiovascular and Diabetes Research, Leeds Institute of Cardiovascular and Metabolic Medicine, University of Leeds, Leeds, United Kingdom; Universita degli Studi Magna Graecia di Catanzaro, ITALY

## Abstract

**Background:**

Magnetocardiography (MCG) is a non-invasive technique used to measure and map cardiac magnetic fields. We describe the predictive performance of a portable prototype magnetometer designed for use in acute and routine clinical settings. We assessed the predictive ability of the measurements derived from the magnetometer for the ruling-out of healthy subjects and patients whose chest pain has a non-ischemic origin from those with ischemic heart disease (IHD).

**Methods:**

MCG data were analyzed from a technical performance study, a pilot clinical study, and a young healthy reference group. Participants were grouped to enable differentiation of those with IHD versus non-IHD versus controls: Group A (70 IHD patients); Group B (69 controls); Group C (37 young healthy volunteers). Scans were recorded in an unshielded room. Between-group differences were explored using analysis of variance. The ability of 10 candidate MCG predictors to predict normal/abnormal cases was analyzed using logistic regression. Predictive performance was internally validated using repeated five-fold cross-validation.

**Results:**

Three MCG predictors showed a significant difference between patients and age-matched controls (*P*<0.001); eight predictors showed a significant difference between patients and young healthy volunteers (*P*<0.001). Logistic regression comparing patients with controls yielded a specificity of 35.0%, sensitivity of 95.4%, and negative predictive value for the ruling-out of IHD of 97.8% (area under the curve 0.78).

**Conclusion:**

This analysis represents a preliminary indication that the portable magnetometer can help rule-out healthy subjects and patients whose chest pain has a non-ischemic origin from those with IHD.

## Introduction

Distinguishing between acute coronary syndrome (ACS) and non-cardiac chest pain is a major challenge in the emergency department (ED) [1]. Current diagnostic tests for patients presenting with chest pain are time-consuming and involve an electrocardiogram (ECG), as well as serial blood biomarker testing [2]. Yet, almost 75% of patients with chest pain of a non-cardiac origin are triaged through this pathway [3–5]. The ability to rule-out ACS earlier would greatly improve patient care and hospital resource utilization [5]. Furthermore, current diagnostics fail to detect up to 2% of non-ST-elevated myocardial infarction (NSTEMI) or unstable angina patients, who are inappropriately discharged from hospital [6,7].

Electrical activity in the heart simultaneously produces a magnetic field [8], which can be detected non-invasively using magnetocardiography (MCG) [9]. MCG is emission-free and of very low risk to the patient [10]. Since magnetic fields are not attenuated by bodily tissue or fluids, MCG is more sensitive than an ECG to very weak cardiac signals [11–13]; MCG can detect ischemia-induced deviations in depolarization and repolarization with greater accuracy than an ECG [14]. Substantial improvements in the diagnosis of ischemic events, such as NSTEMI, which are difficult to detect by an ECG, have already been demonstrated with MCG [13,15].

Superconducting Quantum Interference Device magnetometers have been widely used in cardiology and neurology research [12,13]. However, they require separation from sources of background interference, liquid helium cooling, highly trained operators, and have high running costs. A portable prototype magnetometer was recently designed to overcome the limitations of existing technology [16]. Its novel coil configuration allows high sensitivity detection with negligible background magnetic field interference [16]. The magnetometer can be used at the patient’s bedside, making it suitable for use in acute and routine clinical settings. A technical performance study investigated the predictive performance and reliability of candidate predictors obtained from a broad spectrum of ischemic heart disease (IHD) patients and age-matched healthy volunteers (S1 Text). The results showed an overall good discrimination performance, with generally good to excellent reliability for the derived measurements.

In the current study, we assessed predictive ability of the measurements derived from the magnetometer for the rule-out of healthy subjects and patients whose chest pain has a non-ischemic origin from those with IHD.

## Materials and methods

### Data collection

MCG data were collected from two studies. A technical performance study was conducted in patients (≥25 years) with suspected IHD and in healthy age-matched volunteers (S1 Text). A subgroup of patients with NSTEMI within 3 days of enrollment was also recruited. Patients were recruited from cardiology clinics at Leeds General Infirmary. Patients were identified from medical records and approached by the Clinical Investigator during a clinic appointment or on the hospital ward if the patient was an in-patient. Healthy volunteers were recruited by poster advertising in the cardiology clinic.

A pilot clinical study was conducted in NSTEMI patients (≥18 years) requiring admission for chest pain (troponin ≥50 ng/L 12 h post-onset of chest pain), and in a control group of non-IHD (NIHD) patients experiencing chest pain (ClinicalTrials.gov, number: NCT02359773, available at: https://www.clinicaltrials.gov/ct2/show/NCT02359773). Patients with ST-elevated myocardial infarction, hemodynamic instability, or revascularization were excluded from this cohort. NIHD patients (≥18 years) were eligible if they had a referral due to chest pain in the last 8 weeks and a negative magnetic resonance imaging scan, Myoview, or a stress echocardiogram within 4 weeks of the first MCG scan. Patients with symptoms of ACS or troponin ≥50 ng/L were excluded from this cohort.

Patients with a pacemaker or pregnant/lactating women were excluded. Additional exclusion criteria included: internal cardiac defibrillator or active implantable device; metal implants in the torso; and any comorbidity that prevented patients from being scanned. These studies were approved by the relevant Institutional Review Board and all clinical investigations were conducted according to the principles expressed in the Declaration of Helsinki. Written informed consent was obtained prior to any study-related procedures. The technical performance study was approved by the National Research Ethics Service (NRES) Committee Yorkshire & The Humber—Leeds Central, with Ethics Certificate number 12/YH/0562. The pilot clinical study was registered (protocol NCT02359773) on ClinicalTrials.gov., and approved by the NRES Committee Yorkshire & The Humber—Leeds Central, with Ethics Certificate number 14/YH/1222.

An additional data set was collected from young (<30 years) healthy volunteers as a reference standard for a normal scan. Young healthy volunteers were recruited by poster advertising at the University of Leeds.

Data were grouped to allow differentiation of patients from controls: Group A (IHD patients), Group B (controls), and Group C (young healthy volunteers).

### MCG recordings

Scans were carried out for 10 min in a supine position in an unshielded room. Participants removed upper garments and wore a hospital gown to minimize potential interference. For NSTEMI patients, the scan was conducted within 48 h of the onset of chest pain and before coronary angioplasty or other surgical intervention; for NIHD patients, the scan was completed within 4 weeks of a negative functional test for IHD.

MCG signals were baseline-corrected and averaged, centering on the R wave peak, to increase the signal-to-noise ratio. A custom Python program was used to extract candidate predictor estimates from the MCG data. The signal processing software also performed virtual gradiometry and frequency domain filtering [16]. Ten MCG predictors were measured from the ventricular depolarization phase (S1 Table; S1 Fig) [15,17,18].

### Statistical analysis

Analysis of variance was used to compare the candidate MCG predictors between patients and controls. Regularized logistic regression modeling [19] was employed to study the relationship between the response variable (control vs patient) and the 10 candidate MCG predictors. Model 1 was fitted on all available data with both Group B and Group C as controls. Model 2 included Group B only as a control, and Model 3 included Group C only as a control. The patient group (Group A) was the same across all three models.

Several measures of predictive performance were reported [19]. The discriminatory ability of the models to distinguish patients and controls was evaluated using the area under the receiver operator characteristics curve (AUC). The AUC varies between 0.5 and 1.0, with a higher value indicating better performance [20].

The ability of the models to rule-out subjects was assessed via sensitivity, specificity, and negative predictive value (NPV) using a cut-off on the receiver operator characteristics at which sensitivity exceeded 90%. These are defined in the confusion matrix given in Table 1.

**Table 1:** Confusion matrix for a binary classifier.

	Predicted		
	Positive	Negative	
Positive	TP	FN	Sensitivity = TP/TP+FN
Negative	FP	TN	Specificity = TN/TN+FP

TP = True positive: Diseased individuals correctly diagnosed as sick; FP = False positive: Healthy individuals wrongly predicted as sick; TN = True negative: Healthy individuals correctly predicted as healthy; FN = False negative: Diseased individuals wrongly predicted as healthy.

The NPV is not intrinsic to the test and depend also on the prevalence of disease. Assuming IHD prevalence of 15%, NPV is calculated using the following formula based on Bayes' theorem:

$$NPV=\frac{specificity\times (1-prevalance)}{\left(1-sensitivity)\times prevalance+specificity\times (1-prevalence\right)}$$

The models were internally validated using 10 times repeated 5-fold cross validation with stratified sampling [20], whereby the model was developed on 80% of the data and tested on the remaining 20%. The average performance was calculated over five repetitions and the revised average value was viewed as a more accurate estimate of the model performance.

Statistical analysis was performed using R version 3.2.2 (R Core Team) statistical language. Functions from the glmnet library for logistic regression and cross validation (glmnet and cv.glmnet) were used.

## Results

### Participants

**Fig 1:**
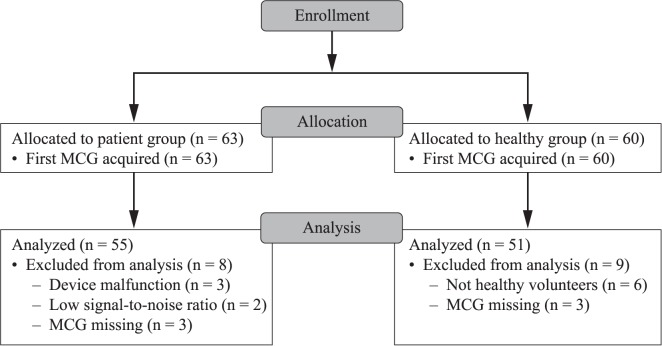
CONSORT Diagram: Technical performance study. Participant flow through the technical performance study. Data were analyzed for 55/63 patients and 51/60 healthy controls.

Between August 2013 and November 2015, 60 patients and 60 healthy volunteers were enrolled in the technical performance study; an additional three patients were recruited to replace scan malfunctions. The mean participant age was 64.2 years (± 10.79) and 37% were female (S2 Table). A total of 55/63 patients and 51/60 healthy volunteers were eligible for analysis (Fig 1). Three patients had all MCG scans lost due to device malfunction, two patients had scans excluded due to low signal-to-noise ratio, and three patient’s scans were all missing. Six healthy volunteers were found to have a history of IHD, and three were missing all their MCG scans.

**Fig 2:**
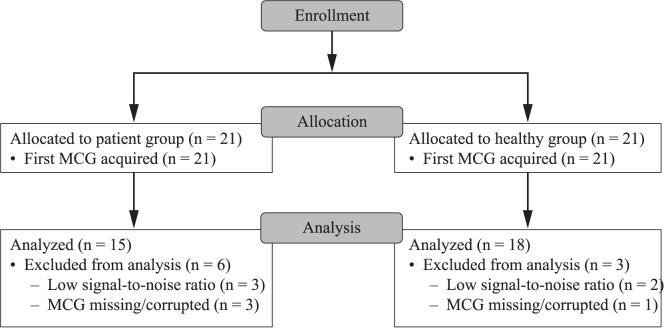
CONSORT Diagram: Pilot clinical study. Participant flow through the pilot clinical study. Data were analyzed for 15/21 patients and 18/21 healthy controls.

Between March 2015 and May 2015, 21 NSTEMI patients and 21 NIHD patients were recruited to the pilot clinical study. The mean patient age was 59.0 years (± 12.71) and 44% were female. A significant between-group difference was noted in gender distribution: 70% of the NIHD patients were female versus 19% of the NSTEMI patients (*P* = 0.002; S3 Table]). Patients with NSTEMI were significantly older than those with NIHD (mean difference –15.5 years; *P*<0.0001). Overall, 15/21 NSTEMI patients and 18/21 NIHD patients were eligible for analysis (Fig 2). Three NSTEMI patients and two NIHD patients had scans excluded due to low signal-to-noise ratio. A further three NSTEMI patients and one NIHD patient were excluded from the analysis due to missing or corrupted MCG data.

### Combined data set

Group A included 70 patients (55 patients from the technical performance study and 15 patients from the pilot clinical study). Group B included 69 controls (51 subjects from the technical performance study with no IHD as confirmed by an ECG and 18 subjects from the pilot clinical study with non-ischemic chest pain and no IHD as confirmed by Myoview or a stress echocardiogram). Group C consisted of 37 young healthy volunteers.

### Candidate MCG predictors

**Fig 3:**
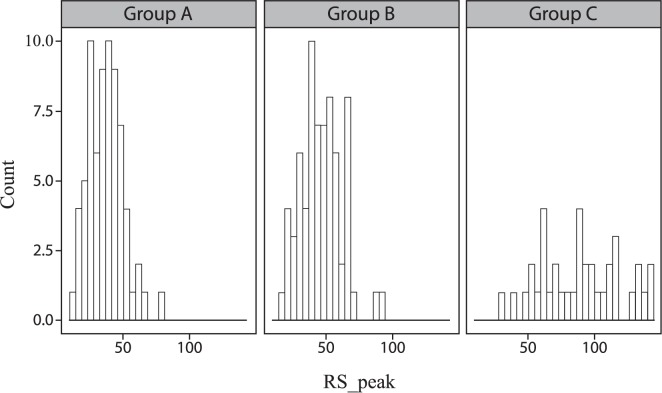
Histogram of the RS_peak predictors. A representative histogram of the RS_peak predictors for study participants enrolled in Group A, Group B, and Group C.

Of the 10 candidate MCG predictors, three showed a significant difference between patients and controls (QR_peak and RS_peak, both *P*<0.01; RS_MMR, *P*<0.05; Table 2). Apart from QR_interval and RS_interval, all of the remaining predictors showed a significant difference between patients and young healthy volunteers. Fig 3 shows a representative histogram of the RS_peak predictors.

**Table 2:** Average value for each predictor for patient and control groups.

Predictor	Group A: patients (n = 70)	Group B: controls (n = 69)	Group C: young healthy volunteers (n = 37)
QR_MMR	1.39 ± 0.53	1.35 ± 0.57	**1.98 ± 0.92++
QR_angle	108.15 ± 15.59	110.78 ± 11.90	**119 ± 8.67++
QR_interval	39.52 ± 5.52	39.49 ± 5.50	37.11 ± 6.97
QR_pd	11.44 ± 1.2	11.31 ± 0.78	**9.63 ± 0.84++
QR_peak	33.88 ± 12.42	**42.03 ± 13.35	**70.44 ± 26.46++
RS_MMR	1.03 ± 0.46	*0.88 ± 0.34	**0.68 ± 0.23++
RS_angle	–67.12 ± 15.02	–63.00 ± 11.44	**–56.68 ± 9.63+
RS_interval	42.14 ± 6.80	42.18 ± 6.21	44.57 ± 8.97
RS_pd	11.48 ± 0.83	11.25 ± 0.74	**9.79 ± 0.95++
RS_peak	37.50 ± 12.76	**46.32 ± 14.94	**88.29 ± 29.52++

* and ** are used for the comparison between Group A and Group B, and Group A and Group C.

+ and ++ are used for the comparison between Group B and Group C. Significance level: +, *P<0.05; ++, **P<0.01.

### Model performance

All logistic regression models showed respectable rule-out ability (Table 3). Model 1 yielded an AUC of 0.82, specificity of 33.0%, sensitivity of 98.6%, and NPV for the ruling-out of patients of 99.3%. A near perfect separation between the groups was achieved in Model 3 comparing patients with young healthy volunteers, with an AUC of 0.96 and specificity of 78.4% (sensitivity and NPV both 100%). Separation between the groups was lower in Model 2, with an AUC of 0.75, specificity of 20.3%, and sensitivity of 94.3% (NPV 95.2%) due to the absence of young healthy volunteers.

**Table 3:** Apparent and cross-validation performance of the logistic regression models.

	Model 1	Model 2	Model 3
Patient group	Group A	Group A	Group A
Control group	Group B + Group C	Group B	Group C
Penalty (alpha)	0.3	0.3	0.3
**Apparent performance**			
AUC	0.82	0.75	0.96
Cut-off	0.20	0.30	0.30
Sensitivity, %	98.6	94.3	100
Specificity, %	33.0	20.3	78.4
NPV, %^a^	99.3	95.2	100
**Cross-validation performance**			
AUC	0.78	0.65	0.94
Cut-off	0.20	0.30	0.30
Sensitivity, %	95.4	91.3	97.3
Specificity, %	35.0	27.6	69.1
NPV, %^a^	97.7	94.7	99.3

AUC, area under the receiver operator characteristic curve; NPV, negative predictive value

Group A, patients; Group B, controls; Group C, young healthy volunteers.

^a^Based on a disease prevalence of 15%.

We noted optimism in the apparent performance of the models compared with the cross-validated performance, which was similar across all models (Table 3).

## Discussion

This study investigated the clinical performance of a portable prototype magnetometer to effectively rule-out patients whose chest pain has a non-ischemic origin from those with IHD. In an ED and an outpatient setting, use of the magnetometer would have the potential to reduce or negate the need for further tests in a subset of these non-ischemic patients.

Based on logistic regression Model 1, using an *a priori* cut-off of 0.2, the magnetometer ruled-out 35.0% of the control group with 97.7% NPV. When utilized as part of the clinical risk stratification, the ability to identify these patients could improve both outcomes and hospital resource utilization.

Although almost 30% of patients presenting with chest pain are aged <50 years, the admission rate for this age group is the lowest amongst all ages [21], highlighting the particular need to differentiate IHD from NIHD and non-cardiac chest pain in this group. Our results showed remarkable discriminating capability of MCG between IHD patients and young healthy volunteers (Model 3), which is in-line with previous studies. Lim et al identified high discriminating capability of MCG predictors between young control and patient groups [15]. In another study, Kandori et al. reported a tendency of the maximum magnetic fields at the QRS complex and T wave to decrease with age [17]. These observations are consistent with an age-related decline in heart function.

The most significant MCG predictors in our analysis were ventricular depolarization predictors. Researchers who focus on depolarization measures report diagnostic performance characteristics of MCG that is substantially equivalent to, if not better than, those who do not [22].

A limitation of our analysis is that we did not consider the effect of gender on MCG. The heterogeneous nature of the IHD patients in the technical performance study could also be a limitation. In the subgroup of NSTEMI patients, MCG scans were recorded up to 3 days after the index event, which could have negatively impacted the sensitivity and NPVs observed. Enrollment of NSTEMI patients to the pilot clinical study relied exclusively on a raised troponin level, the assumption being that troponin is 100% sensitive and specific, which it is not [23,24]. Similarly, recruitment of NIHD patients relied on a normal Myoview or a stress echocardiogram result, neither of which have 100% sensitivity or specificity. Due to the nature of the pilot clinical study it was not possible to implement consecutive recruitment, meaning that selection bias could not be excluded. The healthy young volunteers were recruited from a university campus with an average age <30 years, and thus they did not represent a typical young person presenting to the ED. Finally, the total number of participants analyzed in the study was small. Accurate estimation of the internal validity of a predictive model is problematic when using such a small sample size [20].

In summary, the portable prototype magnetometer fills an unmet need for a rule-out test in triaging chest pain patients to reduce the number of patients with a non-ischemic cause of chest pain who go through screening and follow-up, which could ultimately improve patient care and outcomes, and reduce resource utilization.

## Supporting information

S1 Fig Fifteen averaged overlapped MCG waveforms recorded from a healthy subject. (A) Averaged MCG signal with the ECG superimposed. (B) MFMs at the peaks of the QR and RS section with angle notation. The pole distance and the MFM angle measurements are also visible. ECG: electrocardiogram; MCG: magnetocardiography; MFM: magnetic field map.
(JPG)

S1 Table Definitions of candidate MCG predictors measured from the ventricular depolarization. MCG = magnetocardiography; MFM = magnetic field map.
(DOCX)

S2 Table Baseline demographic characteristics of patients enrolled in the technical performance study. (DOCX)

S3 Table Baseline demographic characteristics of patients enrolled in the pilot clinical study. (DOCX)

S4 Table Confusion matrix for Model 1. (DOCX)

S5 Table Confusion matrix for Model 1 with cross validation. (DOCX)

S6 Table Confusion matrix for Model 2. (DOCX)

S7 Table Confusion matrix for Model 2 with cross validation. (DOCX)

S8 Table Confusion matrix for Model 3. (DOCX)

S9 Table Confusion matrix for Model 3 with cross validation. (DOCX)

S1 Text Unpublished reference. (DOCX)
