# Supplementary figures and images for: A portable prototype magnetometer to differentiate ischemic and non-ischemic heart disease in patients with chest pain
Source: PLoS One. 2018 Jan 19;13(1):e0191241. doi: 10.1371/journal.pone.0191241 (PMC5774725; doi:10.1371/journal.pone.0191241)

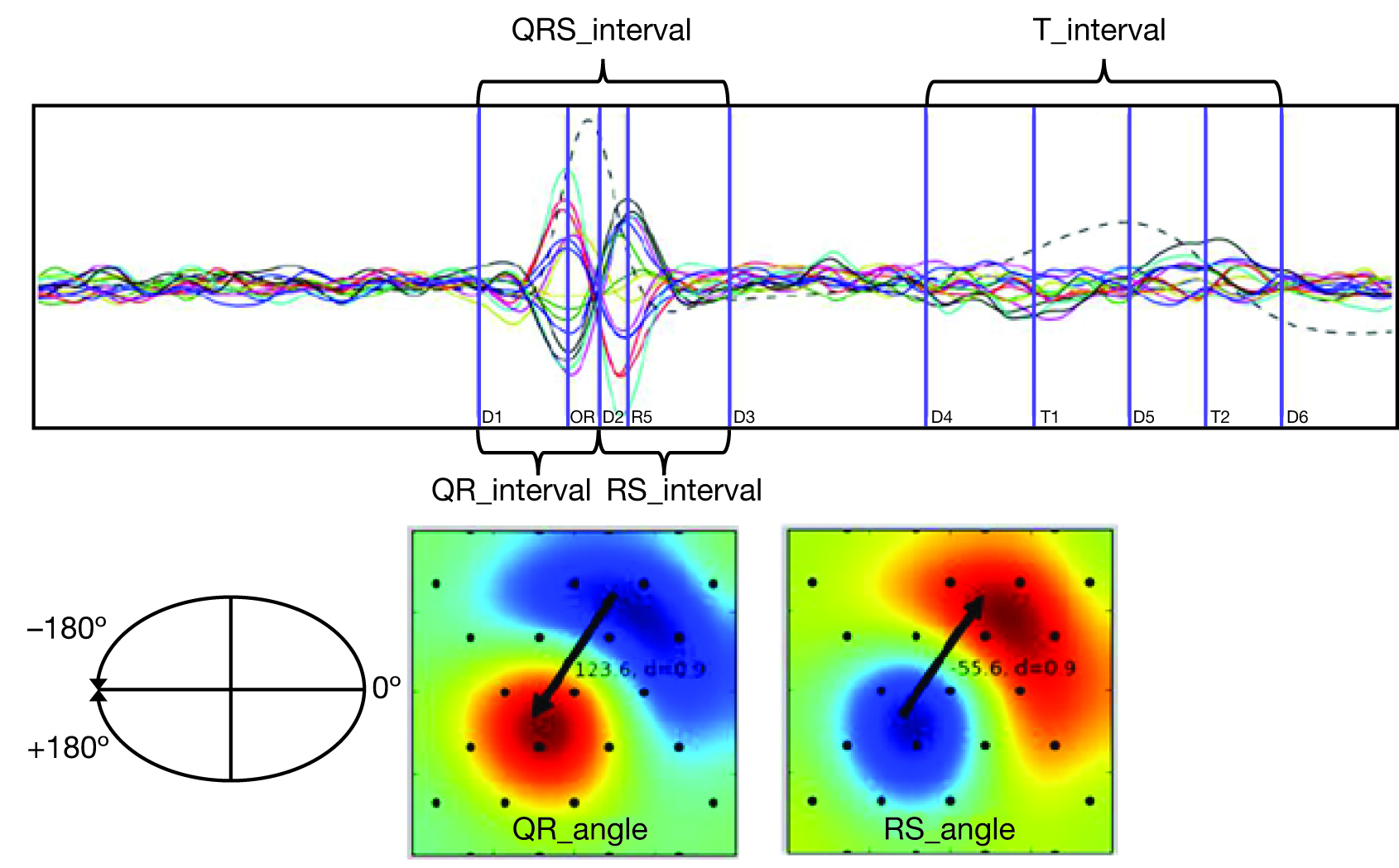

Supplement: S1 Fig — (A) Averaged MCG signal with the ECG superimposed. (B) MFMs at the peaks of the QR and RS section with angle notation. The pole distance and the MFM angle measurements are also visible. ECG: electrocardiogram; MCG: magnetocardiography; MFM: magnetic field map. (JPG) [file pone.0191241.s001.jpg]
