# Supplementary material for: A portable prototype magnetometer to differentiate ischemic and non-ischemic heart disease in patients with chest pain
Source: PLoS One. 2018 Jan 19;13(1):e0191241. doi: 10.1371/journal.pone.0191241 (PMC5774725; doi:10.1371/journal.pone.0191241)
Supplement: S1 Table — MCG = magnetocardiography; MFM = magnetic field map. (DOCX) [file pone.0191241.s002.docx]

**S1 Table. Candidate MCG Predictors Measured from the Ventricular Depolarization Phase.**

| **Amplitude measurements** | **Definition** | **Duration measurements** | **Definition** | **MFM measurements** | **Definition** |
| --- | --- | --- | --- | --- | --- |
| QR_MMR  (no unit) | Amplitude ratio between the negative and positive pole at the QR peak | QR_interval  (ms) | Time duration from the start of the Q wave to the  R peak | QR_angle  (degrees) | Angle between the negative and positive pole at the  QR peak |
| RS_MMR  (no unit) | Amplitude ratio between the negative and positive pole at the RS peak | RS_interval  (ms) | Time duration from the  R peak to the end  of the  S wave | RS_angle  (degrees) | Angle between the negative and positive pole at the RS peak |
| QR_peak  (no unit) | Amplitude difference between the positive and negative pole at the QR peak |  |  | QR_pd | Distance between the negative and positive pole at the QR peak |
| RS_peak  (no unit) | Amplitude difference between the positive and negative pole at the RS peak |  |  | RS_pd | Distance between the negative and positive pole at the RS peak |

MCG, magnetocardiography; MFM, magnetic field map.
