# Supplementary material for: A portable prototype magnetometer to differentiate ischemic and non-ischemic heart disease in patients with chest pain
Source: PLoS One. 2018 Jan 19;13(1):e0191241. doi: 10.1371/journal.pone.0191241 (PMC5774725; doi:10.1371/journal.pone.0191241)
Supplement: S2 Table — (DOCX) [file pone.0191241.s003.docx]

**S2 Table. Baseline Demographic Characteristics of Patients Enrolled in the Technical Performance Study.**

| **Characteristic** | **NSTEMI  (n = 20)** | **IHD  (n = 43)** | **Healthy controls  (n = 60)** |
| --- | --- | --- | --- |
| Age, years | 66 (15.0) | 66 (10.0) | 62 (10.0) |
| Female, n (%) | 10 (50.0) | 10 (23.3) | 26 (43.3) |
| Body mass index, kg/m^2^ | 29.4 (6.3) | 29.0 (4.8) | 27.6 (13.0) |

IHD, **ischemic heart disease**; NSTEMI, **non-ST-elevated myocardial infarction.**

Data are presented as mean (standard deviation), unless otherwise stated.
