# Supplementary material for: A portable prototype magnetometer to differentiate ischemic and non-ischemic heart disease in patients with chest pain
Source: PLoS One. 2018 Jan 19;13(1):e0191241. doi: 10.1371/journal.pone.0191241 (PMC5774725; doi:10.1371/journal.pone.0191241)
Supplement: S3 Table — (DOCX) [file pone.0191241.s004.docx]

**S3 Table. Baseline Demographic Characteristics of Patients Enrolled in the Pilot Clinical Study.**

| **Characteristic** | **NSTEMI  (n = 21)** | **NIHD  (n = 20)** |
| --- | --- | --- |
| Age, years | 66.8 (10.4) | 51.3 (9.8) |
| Female, n (%) | 4 (19.0) | 14 (70.0) |
| Weight, kg | 85.0 (12.9) | 83.7 (21.9) |
| Systolic blood pressure, mm/hg | 132.2 (19.6) | 131.5 (15.9) |
| Diastolic blood pressure, mm/hg | 77.4 (12.5) | 78.9 (9.5) |
| Heart rate, beats/min | 70.1 (10.8) | 72.5 (10.4) |

NIHD, non-**ischemic heart disease**; NSTEMI, **non-ST-elevated myocardial infarction.**

Data are presented as mean (standard deviation), unless otherwise stated.
