# Supplementary material for: A portable prototype magnetometer to differentiate ischemic and non-ischemic heart disease in patients with chest pain
Source: PLoS One. 2018 Jan 19;13(1):e0191241. doi: 10.1371/journal.pone.0191241 (PMC5774725; doi:10.1371/journal.pone.0191241)
Supplement: S4 Table — (DOCX) [file pone.0191241.s005.docx]

**S4 Table. Confusion matrix for Model 1.**

|  | Predicted | |  |
| --- | --- | --- | --- |
|  | Positive | Negative |  |
| Positive | 69 | 1 | Sensitivity = 98.6% |
| Negative | 71 | 35 | Specificity = 33.0% |
