# Supplementary material for: A portable prototype magnetometer to differentiate ischemic and non-ischemic heart disease in patients with chest pain
Source: PLoS One. 2018 Jan 19;13(1):e0191241. doi: 10.1371/journal.pone.0191241 (PMC5774725; doi:10.1371/journal.pone.0191241)
Supplement: S6 Table — (DOCX) [file pone.0191241.s007.docx]

**S6 Table. Confusion matrix for Model 2.**

|  | Predicted | |  |
| --- | --- | --- | --- |
|  | Positive | Negative |  |
| Positive | 66 | 4 | Sensitivity = 94.3% |
| Negative | 55 | 14 | Specificity = 20.3% |
