# Supplementary material for: A portable prototype magnetometer to differentiate ischemic and non-ischemic heart disease in patients with chest pain
Source: PLoS One. 2018 Jan 19;13(1):e0191241. doi: 10.1371/journal.pone.0191241 (PMC5774725; doi:10.1371/journal.pone.0191241)
Supplement: S8 Table — (DOCX) [file pone.0191241.s009.docx]

**S8 Table. Confusion matrix for Model 3.**

|  | Predicted | |  |
| --- | --- | --- | --- |
| True\Predicted | Positive | Negative |  |
| Positive | 70 | 0 | Sensitivity = 100% |
| Negative | 8 | 29 | Specificity = 78.4% |
