# Supplementary material for: A portable prototype magnetometer to differentiate ischemic and non-ischemic heart disease in patients with chest pain
Source: PLoS One. 2018 Jan 19;13(1):e0191241. doi: 10.1371/journal.pone.0191241 (PMC5774725; doi:10.1371/journal.pone.0191241)
Supplement: S9 Table — (DOCX) [file pone.0191241.s010.docx]

**S9 Table. Confusion matrix for Model 3 with cross validation.**

|  | Predicted | |  |
| --- | --- | --- | --- |
| True\Predicted | Positive | Negative |  |
| Positive | 13.6 | 0.38 | Sensitivity = 97.3% |
| Negative | 2.3 | 5.14 | Specificity = 69.1% |
