# Supplementary material for: A portable prototype magnetometer to differentiate ischemic and non-ischemic heart disease in patients with chest pain
Source: PLoS One. 2018 Jan 19;13(1):e0191241. doi: 10.1371/journal.pone.0191241 (PMC5774725; doi:10.1371/journal.pone.0191241)
Supplement: S1 Text — (DOCX) [file pone.0191241.s011.docx]

**S1 Text. Unpublished Reference.**

Al-Shimary A, Ghasemi-Roudsari S, Varcoe B, Brown S, Byrom R, Kearney L, Kearney M. The technical performance of a new portable magnetocardiography device to differentiate ischemic and non-ischemic heart disease in patients with chest pain. Manuscript in preparation.
